# Suboptimal management of central nervous system infections in children: a multi-centre retrospective study

**DOI:** 10.1186/1471-2431-12-145

**Published:** 2012-09-07

**Authors:** Christine Kelly, Aman Sohal, Benedict D Michael, Andrew Riordan, Tom Solomon, Rachel Kneen

**Affiliations:** 1 Neurological Infectious Disease, Brain Infections Group, Institute of Infection and Global Health, University of Liverpool, Liverpool, UK; 2 Paediatric Neurology Registrar, Littlewood Neurosciences Unit, Alder Hey Children’s Hospital NHS Foundation Trust, Liverpool, UK; 3 NIHR Doctoral Research Registrar Brain Infections Group, Institute of Infection and Global Health, University of Liverpool, Liverpool, UK; 4 Division of Neurological Science, Walton Neuro-Centre NHS Foundation Trust, Liverpool, UK; 5 Paediatric Infectious Diseases Department of Infectious Diseases, Alder Hey Children’s Hospital NHS Foundation Trust, Liverpool, UK; 6 Director, Institute for Infection and Global Health, University of Liverpool, Liverpool, UK; 7 Paediatric Neurologist and Honorary Clinical Lecturer Littlewoods Neuroscience Unit, Alder Hey Children’s Hospital NHS Foundation Trust, Liverpool, L12 2AP, UK; 8 Brain Infections Group, Institute of Infection and Global Health, University of Liverpool, Liverpool, UK

**Keywords:** Encephalitis, Meningitis, Central nervous system infection, Aciclovir, Lumbar puncture

## Abstract

**Objective:**

We aimed to audit the regional management of central nervous system (CNS) infection in children.

**Methods:**

The study was undertaken in five district general hospitals and one tertiary paediatric hospital in the Mersey region of the UK. Children admitted to hospital with a suspected CNS infection over a three month period were identified. Children were aged between 4 weeks and 16 years old. Details were recorded from the case notes and electronic records. We measured the appropriateness of management pathways as outlined by national and local guidelines.

**Results:**

Sixty-five children were identified with a median age of 6 months (range 1 month to 15 years). Ten had a CNS infection: 4 aseptic meningitis, 3 purulent meningitis, 3 encephalitis [2 with herpes simplex virus (HSV) type 1]. A lumbar puncture (LP) was attempted in 50 (77%) cases but only 43 had cerebrospinal fluid (CSF) available for analysis. Of these 24 (57%) had a complete standard set of tests performed. Fifty eight (89%) received a third generation cephalosporin. Seventeen (26%) also received aciclovir with no obvious indication in 9 (53%). Only 11 (65%) of those receiving aciclovir had CSF herpes virus PCR. Seventeen had cranial imaging and it was the first management step in 14. Treatment lengths of both antibiotics and aciclovir were highly variable: one child with HSV encephalitis was only treated with aciclovir for 7 days.

**Conclusions:**

The clinical management of children with suspected CNS infections across the Mersey region is heterogeneous and often sub-optimal, particularly for the investigation and treatment of viral encephalitis. National guidelines for the management of viral encephalitis are needed.

## Background

Infections of the central nervous system (CNS) can present with a wide variety of clinical symptoms and signs which are often non-specific, especially in infants and children. Both meningitis and viral encephalitis are neurological emergencies requiring urgent investigation and treatment; [1] especially as distinguishing between the syndromes of meningitis and encephalitis and determining whether the underlying cause is bacterial or viral on clinical grounds is not straightforward. Proven or suspected CNS infections in children are a frequent reason for hospital admission and a significant cause of morbidity and mortality [2,3]. Amongst children less than 14 years of age, UK hospital statistics data recorded 2656 children admitted with a proven CNS infection as their primary diagnosis in the financial year 2008 to 2009. In the same time frame, these children occupied 24,977 bed days [4].

Guidelines for the management of bacterial meningitis and meningococcal disease have been introduced[5,6] but the management of viral CNS infections has been relatively neglected. Despite the significant long term morbidity associated with acute encephalitis in children (reported to occur in over a half of the patients)[7] and the evidence that early diagnosis and treatment of herpes simplex virus (HSV) encephalitis improves outcome,[8] there is comparatively little research into its incidence, clinical features and management in the UK.

An earlier study of children with suspected encephalitis in a tertiary paediatric hospital in our region found that the management was often haphazard [9]. This current study aims to audit the management of children with suspected CNS infection in all hospitals admitting children across the region, including the original tertiary hospital. As far as the authors are aware, this is the first comparative study in children examining the incidence, clinical features and management of acute bacterial and viral CNS infections in a single population.

## Methods

Over a three month period (1^st^ September to 1^st^ December 2007), we undertook a multicentre cross-sectional retrospective cohort study of the clinical case notes and electronic databases of children (aged 1 month to 16 years) admitted with suspected acute CNS infections to six hospitals across the Merseyside region, including five district general hospitals and the specialist children’s hospital (providing secondary care to Liverpool and tertiary care to Merseyside & North Wales) where the pilot study was conducted. The study was approved by each of the Clinical Research and Audit Departments of the individual NHS Trusts involved, and is reported in accordance with STROBE Guidelines (Strengthening the Reporting of Observational studies in Epidemiology; http://www.strobe-statement.org) [10].

### Case inclusion

To identify children in whom a CNS infection might have been suspected we screened doctors ward round registers (lists of all patients admitted to the ward with their final diagnoses), electronic laboratory records to identify patients who had cerebrospinal fluid (CSF) analysis and electronic pharmacy records to identify patients who had received an intravenous third generation cephalosporin, intravenous aciclovir or a combination of both medications. Those receiving these drugs for other indications were excluded. Children having an elective lumbar puncture were also excluded. The case notes of identified children were examined and anonymised data, including whether the admitting doctor had suspected a CNS infection, were recorded on a standardised form by a member of the study team.

Clinical management was compared with the NICE (http://www.NICE.org.uk) guidelines for the management of bacterial meningitis and meningococcal septicaemia, the management of febrile children less than 5 years old and with a regional guideline for the management of suspected encephalitis [6,9,11]. These indicate that children with suspected CNS infections should have an urgent LP, unless they have one of the following contraindications: Glasgow coma score (GCS) <9 or declining, focal neurological signs, strong suspicion of meningococcal septicaemia, systemic shock, bradycardia (heart rate <60 beats per minute), systemic hypertension blood pressure >95^th^ centile for age), signs of raised intracranial pressure, altered pupillary response, abnormal posturing or abnormal respiratory pattern [12]. A cerebrospinal fluid (CSF) pleocytosis was defined as more than 5 white blood cells per millilitre. Data was collected in the local hospitals and entered into an anonymous centralised database in accordance with national guidance.

### Clinical case definitions

For the purposes of analysis, we classified the patients three times during the study. If children were treated with intravenous aciclovir they were classified as having ‘suspected encephalitis’ and if they were only treated with a third generation cephalosporin they were classified as having ‘suspected meningitis’. The second classification was a clinical case definition on the basis of their presenting features, initial CSF and cranial imaging findings (Table 1). Typical imaging findings on CT included hypodensity in the brain parenchyma with associated oedema and on MRI included high signal changes in the brain parenchyma on T2 weighted images with associated oedema [11]. Patients that met the definition of purulent or aseptic meningitis or encephalitis were considered to have a ‘clinically diagnosed CNS infection’ whereas those with meningism or encephalopathy only were not. After microbiological/virological analysis was completed, patients were further classified as having a ‘microbiologically or virologically confirmed CNS infection’, if an appropriate pathogen was identified. For children who met none of the clinical case definitions the most likely diagnosis as judged clinically was used. We defined the ‘time to suspicion of a CNS infection’ as the time from the first medical review to the time the event or intervention was first documented in the case notes.

**Table 1:** Clinical case definitions for classification of children with suspected CNS infection

	**Clinical and initial investigatory criteria**	**Microbiological & virological confirmation**
Encephalopathy only	Altered consciousness with no evidence of typical abnormalities in imaging or CSF analysis (i.e. CSF white cell count <5/ml)	
Encephalitis	Altered consciousness with no other cause identified & evidence of typical abnormalities in imaging or CSF analysis (i.e. CSF white cell count ≥5/ml)	Defined as ‘microbiologically/virologically confirmed' if a pathogen was identified by culture or PCR of the CSF
Meningism only	Meningism (neck stiffness, bulging fontanelle and/or photophobia) without evidence of altered consiousness and no evidence of typical abnormalities in imaging or CSF analysis (ie white cell count <5/ml)	
Purulent Meningitis	Febrile illness or meningism with no altered consciousness & CSF WCC >1000/ml or between 100 and 1000/ml with a predominance of polymorphonuclear cells and a CSF: plasma glucose ratio <0.5 (or unpaired CSF glucose <5 mmol)	Defined as 'microbioloigcally confirmed' if a pathogen was identified by culture or PCR of the CSF or blood
Aseptic meningitis	Febrile illness or meningism with no altered consciousness with a normal CSF: plasma glucose ratio (>0.5) & either a CSF white cell count of 5-20/ml, or 20- 1000/ml with a lymphocyte predominance	Defined as ‘microbiologically/virologically confirmed’ if a pathogen was identified by culture or PCR of the CSF or blood

A ‘full CSF order set’ was defined as CSF being sent for the following investigations: a white cell count (WCC), red blood cell count (RBC), gram stain, glucose level (with paired plasma sample) and protein level.

### Statistics

The t- test and Mann Whitney U test were used to analyse categorical and continuous nonparametric data respectively, with the statistics packages Stats direct and SPSS (SPSS Inc 2009). Odds ratios with 95% confidence intervals were calculated where appropriate with statistical significance defined as p < 0.05.

For epidemiological analysis, the number of children served by the hospitals participating in our study was identified from the published literature where available and where not available was estimated from the Trust catchment population using national population data [13].

## Results

One unit was unable to provide data as there was no record of an LP being performed on a child within the 3 month period and there was no other method of identifying the cases.

### Patients

Sixty five patients were identified. No children who were ultimately diagnosed with a CNS infection had the diagnosis missed at admission. Ten met the clinical case definitions for a diagnosis of a CNS infection with six having a pathogen identified (Table 2). Of the 55 without CNS infection, 46 were classified as encephalopathy, five as meningism and four had non-specific features suggestive of a CNS infection but could not be classified further (Table 1). The median (range) age was 6 months (1 month-15 years):48 (72%) were < three years old; 40 (62%) were male. The median (range) age of children who met the clinical case definition for a CNS infection was 4.2 months (1 month to 7 years) and those who did not was 8 months (1 month to 14 years). One child had recently travelled out of the UK (within Europe). None were suspected of being immunosuppressed.

**Table 2:** Details of microbiology results and management in 10 children with a confirmed diagnosis of CNS infection

**Final diagnosis**	**CSF WCC cells/ml**	**CSF glucose mmol/L (CSF/plasma ratio)**	**CSF PCR**	**Blood cultures**	**Treatment**	**Duration (days)**	**Microbiology/ID advice sought?**
Aseptic Meningitis	8	4.5 (no plasma glucose)	Negative	Negative	cephalosporin	4	Yes
	28	3.9 (no plasma glucose)	Negative	*Escherichia coli*	cephalosporin	3	Yes
	20	2.9 (no plasma glucose)	Not done	Negative	cephalosporin	2	Yes
	130	3.7 (0.62)	Not done	Negative	cephalosporin	21	Yes
Purulent Meningitis	Blood stained	Blood stained	Not done	*Haemophillus influenzae type b*	cephalosporin	9	Yes
					aciclovir	1	
	632	4.7 (0.76)	Negative	Group B *Neisseria meningitidis*	cephalosporin	12	Yes
	112	0.17 (0.03)	Negative	Negative	cephalosporin	7	Yes
Encephalitis	0	2.7 (0.44)	Adeno virus species	Negative	aciclovir	8	No
					cephalosporin	8	
	0	5.2 (no plasma glucose)	Herpes Simplex Virus 1	Negative	aciclovir	7	No
					cephalosporin	7	
	7.2 (lymphocytosis)	3.6 (0.56)	Herpes Simplex Virus 1	Negative	aciclovir	14	Yes
					cephalosporin	2	

The catchment population for these hospitals was 537,620 children giving an approximate annual incidence of: any CNS infection 7.45/100 000; aseptic meningitis 3.0/100 000; purulent meningitis 2.2/100 000 and encephalitis 2.2/100 000.

### Clinical features

Common presenting symptoms for the cohort as a whole were fever (72%) and a prodromal illness (74%). Clinical features alone did not accurately distinguish between children with and without a final diagnosis of CNS infection. Half of the children with a final diagnosis of CNS infection had vomiting or a rash compared to 35% and 27% respectively for children with an alternative final diagnosis (p > 0.05). Thirty percent with a CNS infection had a Glasgow Coma Score (GCS) less than 15 compared to 13% of those without (p > 0.05). Less than 10% of children in either group exhibited neck stiffness, photophobia or focal neurology. The median (range) time to suspicion of CNS infection was one hour (0–60 hours). The median (range) time to suspicion of CNS infection was significantly less for children who were treated with aciclovir compared to those who were only treated with a 3^rd^ generation cephalosporin [0 hours (0 to 60) versus 1 hour (0 to 48) respectively; p value 0.047]. However, the median (range) time to performing the LP was significantly longer for those initially suspected of encephalitis compared to those suspected of meningitis [13.5 hours (0.5 to 72) versus 5.5 hours (1 to 24) respectively; p value 0.036].

### Neurological features

Fifty-six (86%) had an admission GCS documented. It was reduced in 10 (15%); with eight scoring <8. Sixteen (25%) had seizures: 14 (22%) had generalised tonic-clonic seizures prior to admission; five had further seizures in hospital. Two (3%) additional patients had focal seizures after admission. One had a post-ictal Todd’s paresis following an atypical febrile convulsion. One further patient complained of parasthesiae in one arm; his final diagnosis was migraine.

### Management pathways

**Figure 1:**
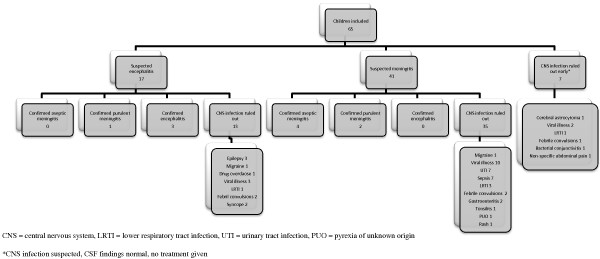
Suspected and final diagnosis for 65 children investigated or treated for suspected CNS infection.

**Figure 2:**
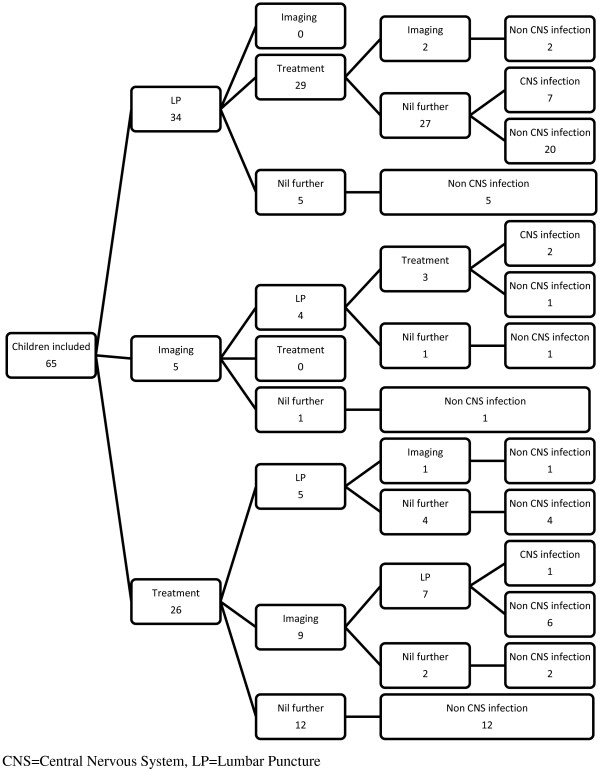
Order of management steps and final diagnosis for 65 children with suspected CNS infection.

Figure 1 demonstrates the final diagnoses for all children compared to their suspected diagnosis at presentation. Management pathways and outcomes are summarised in Figure 2. The first management step was: LP in 34 (52%), treatment first 26 (40%) [17 (65%) a third generation cephalosporin and 9 (15%) a combination of a cephalosporin and aciclovir], cranial imaging five (8%). Figure 1 details the final diagnoses.

### Investigations

#### Lumbar punctures

Overall, an LP was attempted in 50 children (77%). CSF was suitable for analysis in 43 (86%): five had a dry tap and two were heavily blood stained.

Thirty three children (51%) did not have an LP performed at initial presentation. Twelve of these children (18%) were initially deemed to have a contraindication to LP (five had new onset seizures, four had clinical signs of raised intracranial pressure, one had a new hemiplegia, one had hemisensory symptoms, and one had poor skin perfusion). For the remaining 11 children there had been no contra-indication identified.

Following further investigation, 8 out of the 12 children who initially had a contra-indication to LP went on to have an LP attempted. Therefore a total of fifteen children (23%) had no LP attempted at any time. One of these children had a proven contra-indication to LP following investigation (final diagnosis brain tumour) but no obvious reasons were given for not undertaking an LP in the remaining 14 children.

Looking more closely at these 14 children, 4 cultured an organism in blood cultures (Group B *Neisseria meningitidis:* final diagnosis bacterial meningitis, *Staphylococcus aureus:* final diagnosis urinary tract infection, *Streptococcus pyogenes:* final diagnosis septicaemia and *Streptococcus agalactiae:* final diagnosis septicaemia). Another child grew *Haemophilus influenzae* from an endotracheal aspirate and had a final diagnosis of lower respiratory tract infection. The other nine had a short median (range) length of stay of 2.5 days (1 – 3) and had all suffered febrile convulsions.

#### Imaging

Seventeen (26%) children had cranial imaging [12 Computed Tomography (CT) scans, four fontanelle ultrasounds, one magnetic resonance imaging (MRI)]. In 14, this was the first or second management step before LP was considered and 11 went on to have an LP (three had abnormal CSF analysis). Three children had imaging after LP; one child (ultimately diagnosed with a focal epilepsy) had a CT after LP and the remaining 2 children had cranial ultrasound (both diagnosed with viral illness). Two imaging results were abnormal: one brain tumour, one bilateral abnormal fronto-temporal lobe changes on T2 weighted images on MRI (HSV type 1 encephalitis by CSF PCR). Twelve of these 14 had an LP contraindication but for two there was no obvious indication for imaging (final diagnosis was syncope). For the three who did not have an LP after imaging, one had a clear contraindication (tumour), but there was no obvious reason for the other two children. Their blood cultures were positive (see section on LP above).

#### CSF analysis

Of the 43 patients with CSF available for analysis, 24 (57%) had a ‘full CSF order set’ (see methods) of standard investigations performed. All had documentation of the CSF WCC (including differential) and RBC, 39 (93%) had protein recorded, 41 (98%) had glucose recorded but only 26 (62%) had simultaneous blood glucose measured. A CSF pleocytosis was present in nine children with a median (range) of 8 (5–632) cells/ml. Gram staining was negative for all samples. Culture was positive for two; one *Haemophilus influenzae* type b and one *Acinetobacter* species (considered a contaminant). Eighteen (38%) underwent polymerase chain reaction (PCR) analysis for common CNS viruses (HSV types 1&2, adenovirus, enterovirus, varicella zoster and parechovirus) and bacteria (*Neisseria meningitidis* and *Streptococcus pneumoniae*). Eleven (65%) of the seventeen patients treated with aciclovir had viral PCR testing requested. PCR was positive in three (two HSV type 1, one adenovirus). Neither patient with proven HSV encephalitis had a second LP to check for viral clearance after treatment. Of the six who did not have PCR requested, the median (range) length of treatment with aciclovir was 2.5 (1 to 7) days. Table 2 gives details for those children with confirmed CNS infection.

### Treatment

Fifty eight (89%) received treatment (58 third generation cephalosporin, 17 combination of cephalosporin and aciclovir). Twelve (18%) received treatment as their only management step. Seven received no treatment (see details below). The median (range) time to commencing a third generation cephalosporin was 3 (0 to 65) hours and aciclovir was 6 (0 to 72) hours. The median (range) duration of treatment with a cephalosporin was 3 (1 to 21) days and aciclovir was 3.5 (1 to 14) days.

Three of the four children with a final diagnosis of aseptic meningitis were treated with a cephalosporin for less than 7 days. Two children had PCR sent on the CSF for viral pathogens including enterovirus and parechovirus; both were negative (Table 2). The third child, aged one month, had a negative CSF culture but a positive blood culture for *Escherichia coli*. Urine culture was not recorded. She was treated for 21 days with a cephalosporin CSF analysis revealed: WCC 130/ml, protein 0.65 g/l, glucose ratio 62%, CSF culture negative. It is possible this child had purulent meningitis caused by *Escherichia coli* with a comparatively low CSF WCC and normal CSF/plasma glucose ratio or they may have had aseptic meningitis associated with a urinary tract infection, reported previously in infants [14].

Three had a final diagnosis of purulent meningitis (two microbiologically confirmed). One had CSF analysis consistent with purulent meningitis (WCC 112/ml, CSF/plasma glucose ratio 3%, protein 3.8 g/l) but did not have an organism identified on CSF PCR or blood culture; one was positive for *Haemophilus influenzae* type b on blood cultures and CSF PCR and the last patient (CSF WCC 632/ml) grew *Group B Neisseria meningitidis* on blood cultures but not on CSF PCR. These three children were treated for a median (range) of 9 (7 to 12) days. Details are given on Table 2. A fourth child grew *Group B Neisseria meningitidis* on blood cultures and had clinical features of meningitis but did not have an LP.

For the 17 children who were started on aciclovir, 16 had a history of altered consciousness on presentation. Of the three children who went on to have a final diagnosis of encephalitis, two had positive CSF PCR for HSV 1. They were treated with intravenous aciclovir for 7 and 14 days and a third generation cephalosporin for 7 and 2 days respectively. The third child had a positive CSF PCR for adenovirus and was treated with both aciclovir and a third generation cephalosporin for 8 days.

Children with encephalitis tended towards a longer median (range) duration of stay than those with meningitis (aseptic and purulent) [11.5 (8 to 15) and 3 (2 to 9) days respectively].

The median (range) time to LP was significantly longer for those who had it after treatment compared to those who had it before treatment [5 (1 to 49) and 17 (4 to 72) hours respectively; p value 0.0057]. Nine children who had an LP before treatment was started were diagnosed with a CNS infection compared to two who were already on treatment when the LP was performed (p value 0.048). All four children who had an organism identified had the LP performed before starting treatment (p value 0.08).

### Outcome

No children died or required intensive care treatment. None developed renal impairment due to aciclovir. Three were diagnosed with epilepsy. All other patients appeared to have made a good recovery, but full outcome information was not available.

## Discussion

Clinical management of patients with suspected CNS infections across the region is heterogeneous and sub-optimal, particularly for the investigation and treatment of viral encephalitis. In a previous study at the tertiary children’s hospital, we reported that the number of children with suspected CNS infection who had an LP (where no contraindications existed) was only 53% [15]. In this study, of the 64 children who did not have a contra-indication to LP (both before and after imaging), 50 (80%) had an LP attempted. This figure is therefore encouraging and may partially reflect the recent implementation of LP guidelines and junior doctors training sessions at the specialist children’s hospital [15]. However, in those children who had an LP and their CSF was suitable for analysis, a full set of standard investigations was only ordered in 24 (57%) with a paired plasma glucose being the main test missed. This is comparable to the findings in our single site study [9]. Furthermore, no patient who had treatment started before their LP had a bacterium isolated from their CSF. This is similar to previous studies that have demonstrated a reduction in the sensitivity of bacterial CSF culture after antibiotics have been started [16]. It seems possible in this study, that some children treated for suspected CNS infection will not have had a microbiological/virological diagnosis due to not having an LP at all, or failing to order specific tests such as CSF PCR. This makes the ongoing clinical decisions such as when to stop aciclovir or third generation cephalosporins very difficult and children may either be exposed to drugs they do not need and a longer hospital stay, or worse, miss out on necessary treatment.

The management of children started on aciclovir for suspected encephalitis was very variable with the indications for starting aciclovir not always clear. The ideal length of treatment recommended for adults and children with HSV encephalitis is still unknown. Initial trials suggested 10 days [17], but since then, relapses have been reported, so 14 – 21 days is now recommended [18,19]. Additionally, many would recommend repeating the LP to ensure that the CSF is negative for HSV by PCR before stopping treatment and this is supported by a European consensus statement and recently published UK guidelines on the management of children with viral encephalitis [19]. In this study, one child with HSV encephalitis was treated for only seven days and the other for 14. Neither case was discussed with either an infectious diseases or neurology specialist. In addition, neither child had a follow up LP to look for viral clearance.

We found an interesting subset of seven children who were not treated at all following a normal LP and/or cranial imaging. It is known that children with both meningitis and encephalitis can have a normal CSF white cell count in the early stages of the illness [20,21] and in fact two of the children with proven encephalitis in this study had no white cells in the CSF (Table 2). Therefore if a decision is made not to treat a child with enough symptoms or signs to warrant an LP, it would seem sensible to observe them carefully and consider repeating the LP if there is any change in their clinical condition to make sure a CNS infection is not missed. Three of these children were discharged on the day of their LP.

As data was collected retrospectively, it is possible that children with suspected CNS infection in the study time period will not have been included due to incorrect discharge coding or incomplete data to search. One other possible limitation is in the classification of ‘encephalopathy’ (Table 1) as it is recognized that occasionally children with CNS infection may have an acellular CSF in the early stages of their illness [22]. However, none of the children with normal CSF findings had a subsequent clinical course in keeping with a CNS infection. The numbers of patients in the study is small so statistical analysis has been limited and does not allow for comparison between management at the two different types of setting (secondary versus tertiary care).

However, it seems reasonable to conclude that the management of children with suspected CNS infections in our region was very variable and it also seems reasonable to conclude that these findings would be similar in other regions of the UK. Although the percentage of children with suspected CNS infection having an appropriate LP attempted is higher than our previous study, the number of children with a full set of CSF tests requested was disappointing. Over half the children started on aciclovir did not appear to have a clear indication. Of most concern, was the short treatment course for two children with proven HSV type 1 encephalitis. We therefore conclude that national guidelines for the management viral encephalitis are needed.

## Competing interests

The authors declare that they have no competing interests.

## Authors’ contributions

CK analysed the data and drafted the manuscript. AS collected data. BDM designed the study, collected data and reviewed the manuscript. AR Reviewed the manuscript. TS designed the study and reviewed the manuscript. RK designed the study and drafted and reviewed the manuscript. All authors read and approved the final manuscript.

## Disclosure of funding

TS is an MRC senior fellow.

BM is an NIHR doctoral research fellow.

This project was supported by the NIHR Biomedical Research Centre in Microbial Diseases, Liverpool.

## Pre-publication history

The pre-publication history for this paper can be accessed here:

http://www.biomedcentral.com/1471-2431/12/145/prepub
